# Sebacinales Everywhere: Previously Overlooked Ubiquitous Fungal Endophytes

**DOI:** 10.1371/journal.pone.0016793

**Published:** 2011-02-15

**Authors:** Michael Weiß, Zuzana Sýkorová, Sigisfredo Garnica, Kai Riess, Florent Martos, Cornelia Krause, Franz Oberwinkler, Robert Bauer, Dirk Redecker

**Affiliations:** 1 Institut für Evolution und Ökologie, Organismische Botanik, Universität Tübingen, Tübingen, Germany; 2 Botanisches Institut, Universität Basel, Basel, Switzerland; 3 Université de La Réunion, UMR C53 Peuplements végétaux et bioagresseurs en milieu tropical, BP 7151, Saint-Denis, France; Agroscope Reckenholz-Tänikon, Research Station (ART), Switzerland

## Abstract

Inconspicuous basidiomycetes from the order Sebacinales are known to be involved in a puzzling variety of mutualistic plant-fungal symbioses (mycorrhizae), which presumably involve transport of mineral nutrients. Recently a few members of this fungal order not fitting this definition and commonly referred to as ‘endophytes’ have raised considerable interest by their ability to enhance plant growth and to increase resistance of their host plants against abiotic stress factors and fungal pathogens. Using DNA-based detection and electron microscopy, we show that Sebacinales are not only extremely versatile in their mycorrhizal associations, but are also almost universally present as symptomless endophytes. They occurred in field specimens of bryophytes, pteridophytes and all families of herbaceous angiosperms we investigated, including liverworts, wheat, maize, and the non-mycorrhizal model plant *Arabidopsis thaliana*. They were present in all habitats we studied on four continents. We even detected these fungi in herbarium specimens originating from pioneering field trips to North Africa in the 1830s/40s. No geographical or host patterns were detected. Our data suggest that the multitude of mycorrhizal interactions in Sebacinales may have arisen from an ancestral endophytic habit by specialization. Considering their proven beneficial influence on plant growth and their ubiquity, endophytic Sebacinales may be a previously unrecognized universal hidden force in plant ecosystems.

## Introduction

Mutualistic interactions between fungi and plant roots have been a fundamental prerequisite for evolution and biodiversity of land plants. More than 80% of known species of land plants are associated with mutualistic fungi in their roots, facilitating mineral nutrient uptake of the plants [1]. These associations are known as mycorrhizae. There is an amazing morphological and physiological diversity among numerous different types of these plant-fungal interactions. No other fungal group shows a diversity of mycorrhizal types comparable to that found in the Sebacinales, a basidiomycetous order described only recently [2], [3], [4], [5], [6], [7], [8], [9]. DNA sequence analyses have demonstrated a high phylogenetic diversity in this group [2], [7], which is divided into two distinct subgroups, informally designated group A and group B [2]. Though it is known from molecular phylogenetic analyses that Sebacinales belong to the mushroom-forming basidiomycetes (Agaricomycotina) [10], only a few sebacinalean morphospecies producing basidiomes have been described, all of them belonging to group A. Morphological data on group B Sebacinales is very sparse.

Endophytes, as opposed to mycorrhizal or endoparasitic fungi, are commonly defined as fungi colonizing tissues of living plants without formation of detectable interaction structures such as interaction apparatus or arbuscules and without causing disease symptoms on the hosts [11]. Endophytes were shown to occur in all organs of plants [12]. Classic examples are some ascomycetes in grasses which might have gone unnoticed if they did not produce alkaloids having a strong deleterious effect on cattle feeding on the respective grasses [13]. In practice, the separation of mycorrhizal, pathogenic and endophytic habits often seems to be problematic and a better understanding of symbiont interactions is required to refine these definitions. The recent discovery of the beneficial effects of ascomycetous fungal endophytes of the genus *Curvularia* on host plants in geothermal environments [14] has raised considerable interest for endophytes and the mechanisms of their interaction.

Some Sebacinales strains commonly considered as endophytes, particularly the *Piriformospora indica* model strain belonging to group B, have recently been studied intensively, because they significantly enhance plant growth and seed yield, and induce systemic resistance of their host plants against abiotic stress and fungal pathogens [15], [16], [17], [18]. Experimental studies suggest that the fungus improves the nutritional status of its host plants [19], and that programmed death (apoptosis) of cortical cells, which are subsequently densely colonized by *Piriformospora* hyphae, plays an important role in this endophytic interaction [15], [20]. Generally, since some Sebacinales can easily be maintained and propagated without their plant hosts, these strains may be ideal models for the study of beneficial fungus-plant interactions and have a promising perspective for application in sustainable horticulture and agriculture [15], [17], [18], [21], [22].

Sequences of Sebacinales have been sporadically detected by PCR/cloning approaches from herbaceous plants and soil [18], [23]. Sebacinalean fungi other than the strains used in experimental studies were recently detected in a preliminary study based on a few environmental plant samples [24]; these authors raised the question whether Sebacinales may occur as endophytes in the field more frequently than previously thought. Here we address this question and show that these fungi are indeed found as endophytes within plants and that they are ubiquitous.

## Results and Discussion

**Figure 1:**
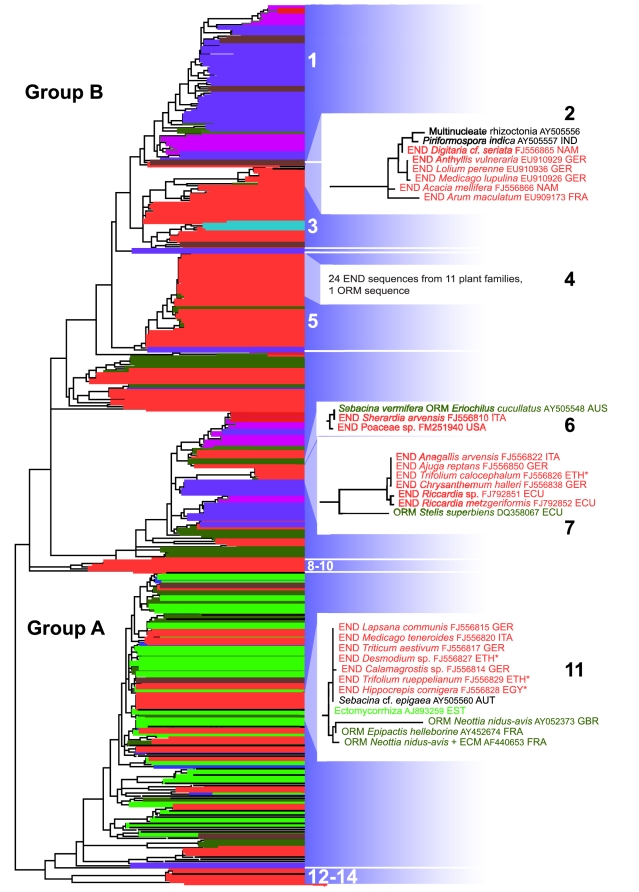
Phylogenetic relationships of Sebacinales based on maximum likelihood analysis of partial nuclear-encoded ribosomal large subunit sequences. Sequences are color-coded by type of symbiosis or origin. Highlighted clades 1-14, including zoomed-in lineages 2, 6, 7 and 11, are explained in the text. Sequences marked with an asterisk (clades 7, 11) are from herbarium specimens collected by G.W. Schimper and T. Kotschy in pioneering field trips in the 1830/40s. The full tree, including all host plants, places of origin, bootstrap values, accession numbers and clades/sequences discussed in the text is shown in Fig. S2, using the same colors and three-letter symbiosis-identifying codes. Red: sequences of endophytes (END), magenta: cavendishioid mycorrhiza (CAV), blue: ericoid mycorrhiza (ERM), dark green: orchid mycorrhiza (ORM), turquoise: jungermannoid mycorrhiza (JMM), bright green: ectomycorrhiza (ECM), brown: soil samples, black: sequences from fruitbodies or cultures. Country codes used here: AUT, Austria; ECU, Ecuador; EGY, Egypt; ETH, Ethiopia; FRA, France; GER, Germany; GBR, Great Britain; ITA, Italy; NAM, Namibia; USA, United States of America.

We analyzed 128 root samples from phylogenetically and ecologically diverse plants from 27 families from four continents and studied the phylogenetic distribution of their sebacinalean endophytes. We used diagnostic group-specific PCR primers for nuclear-encoded rDNA regions designed for this study to detect members of Sebacinales groups A and B and molecular phylogenetics to assess their evolutionary relationships. In addition, we analyzed selected specimens by transmission electron microscopy for the presence of fungal hyphae in the roots with septal pore structures typical for the Sebacinales. This combination of methods is crucial; diagnostic PCR and sequencing alone cannot definitely exclude sebacinalean soil fungi only present on the root surface, and ultrastructural analysis alone cannot discriminate Sebacinales from certain other groups of Agaricomycotina that exhibit a similar septal pore structure. We classify sebacinalean sequences from plants known to be non-mycorrhizal or hosts of arbuscular mycorrhiza, which is known to exclusively involve fungi from the phylum Glomeromycota, as endophytic (Fig. 1).

Molecular analysis yielded 135 sebacinalean nuclear LSU sequences from 128 root samples. We found sebacinalean endophytes in all examined plant families, which span a broad phylogenetic range, from liverworts to Asteraceae (Table S1). Our PCR variants 2 and 3, which involve Sebacinales-specific primers designed for the present study, allow to contiguously amplify and analyze two key rDNA regions (ITS+5.8S and the D1-D2 regions of the nuc LSU; see Fig. S1) presently used for molecular identification and phylogenetic reconstruction employed in the fungi. We even succeeded to amplify Sebacinales sequences from herbarium specimens collected in the 1830-1840s by G.W. Schimper and T. Kotschy on their pioneering botanical excursions to North Africa (FJ556825-30, FJ556857; Fig. S2: 15a,b; 16-20), and were able to sequence the type material of *Sebacina vermifera* from the 1960s (Fig. S2: 21). DNA sequences obtained with these PCR variants can be analyzed both in the context of fungal barcoding (using the ITS portion) and for constructing phylogenetic trees that span both Sebacinales group A and group B or even Fungi as a whole (using the LSU portion). Such data is therefore particularly applicable for analyses of Sebacinales communities using recently developed methods that combine phylogenetic and ecological approaches [25], [26].

**Figure 2:**
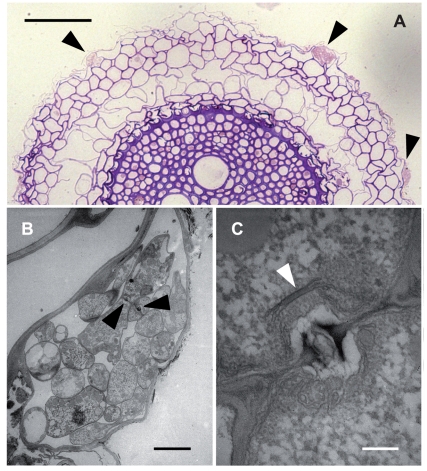
Anatomy and ultrastructure of a field-collected root sample of wheat (*Triticum aestivum*) infested with a sebacinalean endophyte. (*A*) cross section through the root as seen in the light microscope; singular rhizodermal cells are heavily colonized by fungal hyphae (arrowheads). Bar  = 100 µm. (*B*) transmission electron micrograph showing that the colonized rhizodermal cell is dead: intracellular fungal hyphae are not surrounded by host plasma membrane; arrowheads point to hyphal septa in cross section showing septal pores. Bar  = 3 µm. (*C*) dolipore with continuous parenthesome as typical for members of the Sebacinales (arrowhead). Bar  = 200 nm.

Evidence that Sebacinales were in fact present within the plant roots and not just in the rhizosphere was obtained by electron microscopy (Fig. 2). We found that fungal hyphae colonized few dead cortical cells of their host's fine roots, nearly completely filling the host cells. This is well compatible with results obtained from *in vitro* experiments with *P. indica* and the model plants barley and *Arabidopsis* [15], [20]. The colonizing hyphae had the septal pore structure typical for the Sebacinales (Fig. 2).

An overview of the phylogenetic tree of all Sebacinales nuc LSU sequences currently available in GenBank together with our new endophytic sequences is shown in Fig. 1 (for the full tree see Fig. S2). The endophyte sequences were placed in both Sebacinales subgroups A and B, however they were not evenly distributed across these two groups, as most endophytic sequences were placed in group B (Fig. 1). Within group B some clades were dominated by endophyte sequences (Fig. 1: e.g., 3, 5); on the other hand, there are other clades that at this time nearly completely lack known endophytic sequences (Fig. 1: 1). The most basal clades in groups A and B (Fig. 1: 8–10, 12–14) were endophytic; we therefore hypothesize that the endophytic habit may be ancestral in the Sebacinales and the starting point for the development towards specialized mycorrhizal symbioses. However, the close relationship of endophytes, in particular with orchid mycorrhizal strains, could also be indicative of the capability of Sebacinales strains to switch between symbioses, or to fall back on the endophytic habit if no appropriate mycorrhizal partner is present.

Our analyses demonstrate that Sebacinales with closely related or even identical LSU sequences can be found in geographically distant areas (Fig. 1: 7, 11; Fig. S2: 22). The absence of any obvious geographical patterns suggests efficient dispersal. Diverse sequences could be found in the same field site, even in the root system of the same plant host specimen (Fig. S2: 23a-d; 15a,b; 24-26a-b). Host specificity seems to be low as identical sequences were often found across different hosts (Fig. S2: 7, 11). According to present knowledge, sebacinalean fungi involved in ectomycorrhiza (ECM), ectendomycorrhiza (arbutoid mycorrhiza; EEM), and mycorrhizas with heterotrophic or mixotrophic orchids (ORM) have only been found in group A, while ericoid (ERM) and cavendishioid (CAV) mycorrhizas are only known from group B [2], [7]. This is widely confirmed by the present study. Since links between ORM and ECM as well as between EEM and ECM via the same fungus seem to be common [5], [7], it has been hypothesized that all group A Sebacinales are ectomycorrhizal. It was thus surprising to detect endophytic group A Sebacinales (Fig. S2: 12, 27) in meadows where known ECM hosts were missing. Thus, ectomycorrhizal activity is probably not an obligate feature for group A Sebacinales.

In addition to linking ECM to ORM and to EEM, it is likely that Sebacinales are able to connect other mycorrhizal types. We detected the same nuclear LSU sequence in roots of *Sherardia* (Rubiaceae) from Italy and in an Australian orchid (Fig. S2: 6). Other identical sequences were found in an ericad (*Vaccinium*) and a liverwort (*Riccardia*; Fig. S2: 28) or in another ericad (*Cavendishia*), *Melittis* (Lamiaceae) and *Calamagrostis* (Poaceae; Fig. S2: 29). Considering also the numerous cases in which the same nuclear LSU sequence of sebacinalean endophytes was detected in distinct plant species (e.g., Fig. 1: 7, 11), we hypothesize that Sebacinales play a crucial role in connecting individual plants in terrestrial ecosystems across mycorrhizal types. However, nutrient transfer studies are necessary to elucidate this issue.

On the basis of our molecular phylogenetic analysis we can link some of the endophyte sequences to morphospecies (Fig. 1: 6, 13). However, *S. vermifera* strains mostly originating from roots of Australian terrestrial orchids [27] are scattered all over group B, demonstrating the limited usefulness of morphospecies in this context. In fact, from the high genetic distances between specimens that have all been assigned to *S. vermifera* we conclude that much of the huge biodiversity in Sebacinales group B is covered by cryptic species that lack macroscopic fruiting bodies.

Most experimental studies in the Sebacinales have been conducted using the asexual model strain *Piriformospora indica*, which was originally isolated from soil of the Indian Thar desert [28]. Our study reveals that *P. indica* belongs to a well-supported group of closely related endophytic species, in our study represented by sequences from Western European and Namibian Fabaceae, Poaceae, or Araceae (Fig. 1: 2). In view of the biodiversity of endophytic Sebacinales still to be expected (Fig. S3) there is a huge resource of strains that are potentially useful for plant cultivation. Recent studies have shown that the available strains of *S. vermifera* and *P. indica* differ quantitatively in the plant-beneficial effects they induce in different hosts [15], thus the vast genetic diversity of plant-associated Sebacinales offers interesting perspectives for future experimental research and inoculum development.

We show that *Arabidopsis thaliana* is associated with Sebacinales under natural conditions (Fig. S2: 30). This model species belongs to the *Brassicaceae*, which have widely been believed to lack mycorrhizal interactions. Our finding gives new practical relevance to experimental studies on endophytic interactions between *Piriformospora* and the model plant *A. thaliana* [29], [30], since Brassicaceae contain many economically important plants such as cabbage and rape. The presence of sebacinalean endophytes in *Triticum* (Fig. S2: 25a,b; Fig. 2) as well as in *Zea mays* (Fig. S2: 31–33) collected in the field is equally important. Given the positive effects that sebacinalean fungi had on growth, yield and resistance against abiotic stress and fungal pathogens of their plant hosts in experiments under controlled conditions [15], [17], these results underline the feasibility of applying Sebacinales as biological fertilizers and biocontrol agents for arable crops in the future. At the same time, however, our findings imply that inoculated fungi have to be sufficiently competitive against diverse local Sebacinales communities already present in roots and soil.

Since the database of Sebacinales sequences on a worldwide scope is still too sparse, we are currently unable to answer detailed questions on biogeography of this group. Also, since for the present study we merged sequences obtained with different PCR approaches (see the Materials section) and did not rigorously clone all PCR products that could not be sequenced directly, we cannot address the abundance of Sebacinales in more details here. These issues will be dealt with in subsequent, geographically more restricted studies using extended sample numbers.

The ubiquity and diversity of sebacinalean endophytes shown in this study emphasize a previously unrecognized aspect of the plant interactions of this fungal group. Sebacinalean endophytes are not isolated phenomena, but extremely common and potentially important hidden players in plant ecosystems, which may have given rise to the large diversity of mycorrhizal symbioses the Sebacinales participate in. These findings are both relevant for applied research as well as for basic research on the role of Sebacinales in ecosystem functioning and possible shaping of plant communities.

## Materials and Methods

### Sampling

Root samples were taken from various sites in Germany, Switzerland, France, Italy, Austria, Slovenia, Great Britain, the United States, Ecuador, Ethiopia, Namibia, South Africa, and Iceland. Roots were either frozen in liquid nitrogen and stored at −80°C or washed with tap water and herbarized prior to DNA extraction. Sequences FJ556825-30 and FJ556857 (marked with * in Fig. 1, Fig. S2 and Table S1) were obtained from roots of herbarium vouchers sampled by G.W. Schimper and T. Kotschy on their pioneering field trips to North Africa in the 1830s and 1840s. These vouchers are stored in the herbarium at Tubingen University (TUB).

### DNA extraction, PCR, and sequencing

The roots were ground in liquid nitrogen using a micropestle, or in a mixer mill (Retsch, Germany). DNA was extracted with a DNeasy Plant Kit (QIAGEN) according to the manufacturer's instructions, or using an SDS protocol [31]. To selectively amplify Sebacinales DNA we used nested PCRs involving three variants of primer combinations (see Fig. S1): (1) ITS1F [32]/TW14 [33], followed by ITS2Seb [8]/NL4 [34]; (2) NS13 [35]/NLSeb2R (5′-GCCCACTAGAAACTCTCACC-3′), followed by ITS1F/NLSeb1R (5′-CCGCACAAGGCTGATAA-3′); (3) NSSeb1 (5′-CTTCTTAGAGGGACTGTCAGGA-3′)/NLSeb2R, followed by ITS1F/NL4. Variants 2 and 3, for which we developed Sebacinales-specific primers, allow for amplifying and contiguously sequencing the ITS1-5.8S-ITS1 together with the D1/D2 regions of the nucLSU repeat, i.e., the standard regions used in molecular phylogenetic studies and for fungal barcoding. Success of the PCR experiments was checked using agarose gel electrophoresis. The PCR products were purified using a QIAquick PCR purification kit (QIAGEN), a High Pure Kit (Hoffman LaRoche), or by enzymatic purification using EXO-SAP-IT (USB Europe). Purified PCR products were sequenced in both directions using an ABI PRISM Dye-Terminator Cycle Sequencing Kit (Applied Biosystems) and an automated sequencer ABI3130xl, either directly, or after cloning into a vector (TOPO TA, Invitrogen, or pGEM-t, Promega/Catalyse). Sequences were assembled using Sequencher (Gene Codes, Ann Arbor, MI). Taxonomic assignment of the retrieved sequences to the Sebacinales was done by using BLAST [36], [37] against the nucleotide collection of the National Center for Biotechnology Information (NCBI, GenBank; www.ncbi.nlm.nih.gov). The final sequences have been deposited in the NCBI nucleotide collection under the accession numbers EU909214-16, EU909218-19, EU909221, EU909223, EU909225, EU909229, EU910898-03, EU910906-07, EU910910-12, EU910914, EU910917-37, EU910939, FJ556805-11, FJ556814-41, FJ556843-68, FJ792843-44, FJ792846-52, FM251923, FM251925-45, HM030724 (see Table S1).

### Phylogenetic Analysis

A reference dataset was assembled from nuc LSU sequences published in GenBank. To retrieve the full scope of available sebacinalean nuc LSU sequences we used BLAST searches with various query sequences representatively sampled from a recently published molecular phylogenetic analysis [7]. The original dataset was then gradually pruned using preliminary trees produced with MAFFT [38] and RAxML [39] by reducing sets of sequences with identical LSU from identical host plant species to one representative sequence each. The resulting sequence set was complemented with LSU sequences from *Auricularia auricula-judae* and *Trechispora farinacea* as outgroup sequences. The sequences of endophytic Sebacinales determined for this study were added to this set of reference sequences. We aligned the full-length sequences using the ‘localpair’ option in MAFFT, then restricted the sequences to the nuc LSU D1/D2 region, and realigned them again. New sequences positioned on long branches in preliminary phylogenetic trees were checked for the presence of chimera artifacts by blasting anterior and posterior halves of the sequences, respectively, against GenBank. Molecular phylogenetic relationships were estimated using the maximum likelihood method [40] as implemented in RAxML; here, a bootstrap analysis [41] was done on 5000 resampled alignments, and every 5th bootstrap tree was used as a starting point for heuristic maximum likelihood analysis of the original alignment.

### Rarefaction Analysis

We partitioned the endophytic Sebacinales sequences into sequence types by assembling them into contigs using Sequencher; sequences with a D1/D2 minimum match of 99% were treated as representatives of the same sequence type. We then used EstimateS [42] to compute a sample-based analytical rarefaction curve, including confidence intervals based on 1000 replications, treating sequences as samples and sequence types as equivalents of species.

### Microscopy

The ultrastructure was studied with a Zeiss EM 109 transmission electron microscope at 80 kV. Samples were fixed overnight with 2% glutaraldehyde in 0.1 M sodium cacodylate buffer (pH 7.2) at room temperature. Following six transfers in 0.1 M sodium cacodylate buffer, samples were postfixed in 1% osmium tetroxide in the same buffer for 1 h in the dark, washed in distilled water, and stained in 1% aqueous uranyl acetate for 1 h in the dark. After five washes in distilled water, samples were dehydrated in acetone, using 10 min changes at 25%, 50%, 70%, 95%, and 3 times in 100% acetone. Samples were embedded in Spurr's resin [43] and sectioned with a diamond knife. Serial sections were mounted on formvar-coated, single-slot copper grids, stained with lead citrate at room temperature for 5 min, and washed with distilled water. For semi-thin sections, the embedded samples were sectioned, transferred to a microscope slide, stained with new fuchsin and crystal violet, mounted in Entellan (Merck, Germany) under a cover slide, and studied by light microscopy at various magnifications.

## Supporting Information

Figure S1 Map of primers used for PCR and sequencing in the present study. The map is based on GenBank sequences AY505557 and DQ520096, primer lengths are not drawn to scale. The ruler gives number of base pairs, starting from the 5′ end of primer NS13.
(PDF)

Figure S2 Phylogenetic relationships of Sebacinales based on maximum likelihood analysis of partial nuclear-encoded ribosomal large subunit sequences. Color boxes indicate type of symbiosis. Sequences from fruitbodies or cultures that can be assigned to morphospecies are in bold. Circled numbers highlight sequences or clades that are mentioned in the article text. Sequences marked with an asterisk are from herbarium specimens collected in the 1830/40s by G.W. Schimper and T. Kotschy. Numbers on branches are bootstrap support values obtained from 5000 replicates (only values ≥50% are shown), branch lengths are scaled in terms of the number of expected substitutions per nucleotide.
Red: sequences of endophytes (END), magenta: cavendishioid mycorrhiza (CAV), blue: ericoid mycorrhiza (ERM), dark green: orchid mycorrhiza (ORM), turquoise: jungermannoid mycorrhiza (JMM), bright green: ectomycorrhiza (ECM), brown: soil samples.
Country codes: ARG, Argentina; AUS, Australia; AUT, Austria; CAN, Canada; CHL, Chile; CHN, P. R. China; ECU, Ecuador; EGY, Egypt; EST, Estonia; ETH, Ethiopia; FRA, France; GER, Germany; GBR, Great Britain; GUA, Guadeloupe; GUY, French Guyana; ICE, Iceland; IND, India; ITA, Italy; MEX, Mexico; NAM, Namibia; NOR, Norway; REU, Reunion; SAF, South Africa; SLO, Slowenia; SPA, Spain; SUD, Sudan; SUI, Switzerland; THA, Thailand; USA, United States of America.
(PDF)

Figure S3 Analytical sample-based rarefaction curve of endophytic Sebacinales, derived from partial nuclear-encoded ribosomal large subunit sequences. Sequences are treated as samples, sequences of ≥99% similarity were assigned to the same sequence type. Confidence intervals are based on 1000 replicates. As the curve is still far from its saturation level, many other sequence types may be detected by further sampling.
(PDF)

Table S1 Host plants from which endophytic Sebacinales were sequenced for this study and assignment to plant families. Sequences marked with an asterisk are from herbarium specimens collected in the 1830/40s by G.W. Schimper and T. Kotschy.
(XLS)
