# Supplementary figures and images for: Sebacinales Everywhere: Previously Overlooked Ubiquitous Fungal Endophytes
Source: PLoS One. 2011 Feb 15;6(2):e16793. doi: 10.1371/journal.pone.0016793 (PMC3039649; doi:10.1371/journal.pone.0016793)

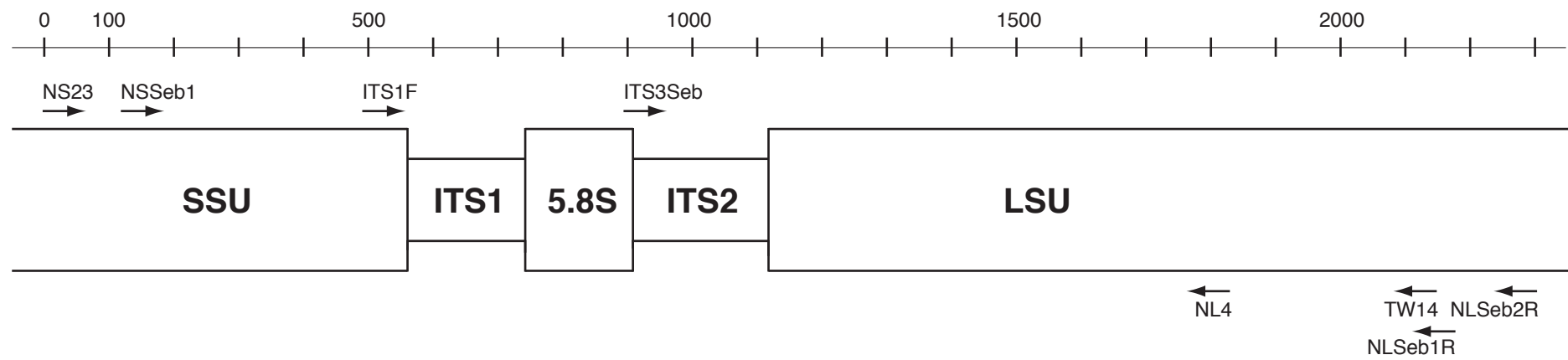

Fig. S1

Supplement: Figure S1 — Map of primers used for PCR and sequencing in the present study. The map is based on GenBank sequences AY505557 and DQ520096, primer lengths are not drawn to scale. The ruler gives number of base pairs, starting from the 5′ end of primer NS13. (PDF) [file pone.0016793.s001.pdf]

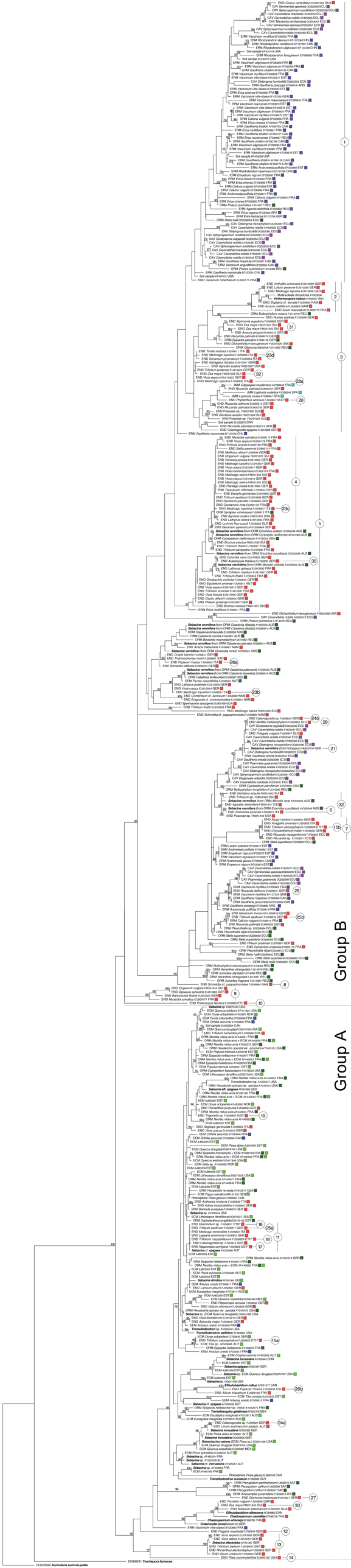

Group B

Group A

Fig. S2

Supplement: Figure S2 — Phylogenetic relationships of Sebacinales based on maximum likelihood analysis of partial nuclear-encoded ribosomal large subunit sequences. Color boxes indicate type of symbiosis. Sequences from fruitbodies or cultures that can be assigned to morphospecies are in bold. Circled numbers highlight sequences or clades that are mentioned in the article text. Sequences marked with an asterisk are from herbarium specimens collected in the 1830/40s by G.W. Schimper and T. Kotschy. Numbers on branches are bootstrap support values obtained from 5000 replicates (only values ≥50% are shown), branch lengths are scaled in terms of the number of expected substitutions per nucleotide. Red: sequences of endophytes (END), magenta: cavendishioid mycorrhiza (CAV), blue: ericoid mycorrhiza (ERM), dark green: orchid mycorrhiza (ORM), turquoise: jungermannoid mycorrhiza (JMM), bright green: ectomycorrhiza (ECM), brown: soil samples. Country codes: ARG, Argentina; AUS, Australia; AUT, Austria; CAN, Canada; CHL, Chile; CHN, P. R. China; ECU, Ecuador; EGY, Egypt; EST, Estonia; ETH, Ethiopia; FRA, France; GER, Germany; GBR, Great Britain; GUA, Guadeloupe; GUY, French Guyana; ICE, Iceland; IND, India; ITA, Italy; MEX, Mexico; NAM, Namibia; NOR, Norway; REU, Reunion; SAF, South Africa; SLO, Slowenia; SPA, Spain; SUD, Sudan; SUI, Switzerland; THA, Thailand; USA, United States of America. (PDF) [file pone.0016793.s002.pdf]

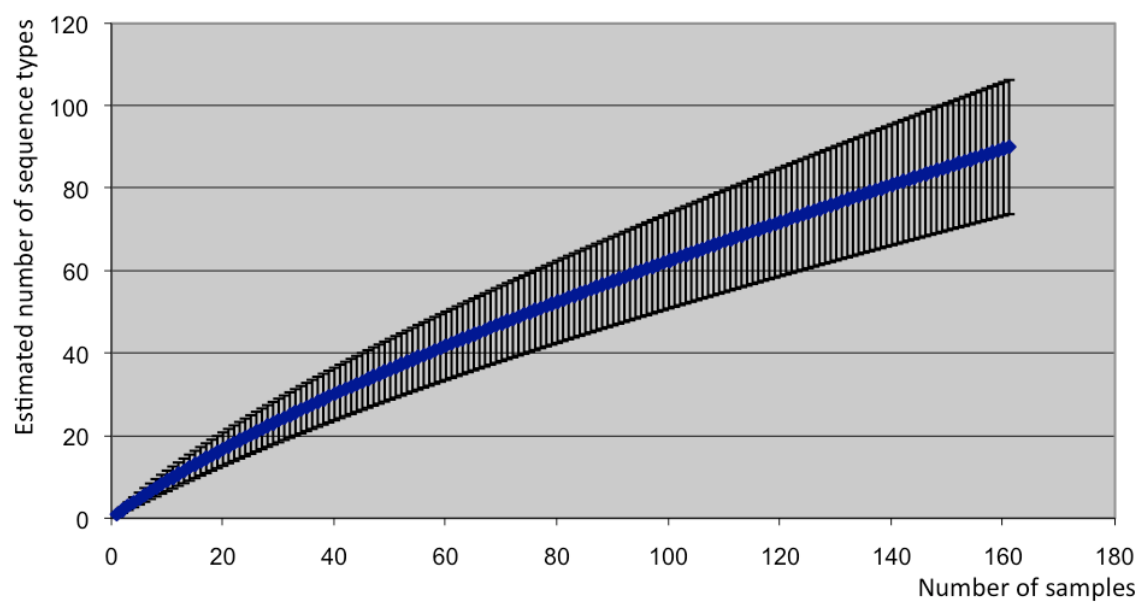

Fig. S3

Supplement: Figure S3 — Analytical sample-based rarefaction curve of endophytic Sebacinales, derived from partial nuclear-encoded ribosomal large subunit sequences. Sequences are treated as samples, sequences of ≥99% similarity were assigned to the same sequence type. Confidence intervals are based on 1000 replicates. As the curve is still far from its saturation level, many other sequence types may be detected by further sampling. (PDF) [file pone.0016793.s003.pdf]
